# Rivers, not refugia, drove diversification in arboreal, sub‐Saharan African snakes

**DOI:** 10.1002/ece3.7429

**Published:** 2021-05-01

**Authors:** Kaitlin E. Allen, Eli Greenbaum, Paul M. Hime, Walter P. Tapondjou N., Viktoria V. Sterkhova, Chifundera Kusamba, Mark‐Oliver Rödel, Johannes Penner, A. Townsend Peterson, Rafe M. Brown

**Affiliations:** ^1^ Department of Ecology and Evolutionary Biology University of Kansas Lawrence KS USA; ^2^ Biodiversity Institute University of Kansas Lawrence KS USA; ^3^ Department of Biological Sciences University of Texas at El Paso El Paso TX USA; ^4^ Laboratoire d’Hérpétologie, Département de Biologie Centre de Recherche en Sciences Naturelles Lwiro Democratic Republic of Congo; ^5^ Museum für Naturkunde – Leibniz Institute for Evolution and Biodiversity Science Berlin Germany; ^6^ Chair of Wildlife Ecology and Management University of Freiburg Freiburg Germany

**Keywords:** historical demography, machine learning, paleo‐distributions, phylogenomics, *Toxicodryas*

## Abstract

The relative roles of rivers versus refugia in shaping the high levels of species diversity in tropical rainforests have been widely debated for decades. Only recently has it become possible to take an integrative approach to test predictions derived from these hypotheses using genomic sequencing and paleo‐species distribution modeling. Herein, we tested the predictions of the classic river, refuge, and river‐refuge hypotheses on diversification in the arboreal sub‐Saharan African snake genus *Toxicodryas*. We used dated phylogeographic inferences, population clustering analyses, demographic model selection, and paleo‐distribution modeling to conduct a phylogenomic and historical demographic analysis of this genus. Our results revealed significant population genetic structure within both *Toxicodryas* species, corresponding geographically to river barriers and divergence times from the mid‐Miocene to Pliocene. Our demographic analyses supported the interpretation that rivers are indications of strong barriers to gene flow among populations since their divergence. Additionally, we found no support for a major contraction of suitable habitat during the last glacial maximum, allowing us to reject both the refuge and river‐refuge hypotheses in favor of the river‐barrier hypothesis. Based on conservative interpretations of our species delimitation analyses with the Sanger and ddRAD data sets, two new cryptic species are identified from east‐central Africa. This study highlights the complexity of diversification dynamics in the African tropics and the advantages of integrative approaches to studying speciation in tropical regions.

## Graphical abstract

We tested the predictions of the classic river, refuge, and river‐refuge hypotheses of tropical rainforest evolution on diversification in the arboreal Sub‐Saharan African snake genus *Toxicodryas*. We used dated phylogeographic inferences, population clustering analyses, demographic model selection, and paleo‐distribution modeling to conduct a phylogenomic and historical demographic analysis of this genus. Our demographic analyses supported the interpretation that rivers have represented strong barriers to gene flow among populations since their divergence, and we found no support for a major contraction of suitable habitat during the last glacial maximum, allowing us to reject both the refuge and river‐refuge hypotheses in favor of the river barrier hypothesis.

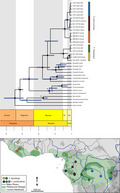

## 1 INTRODUCTION

Three major allopatric diversification mechanisms have been proposed in the classical literature to explain species diversity in the tropics. The “river hypothesis” in which species and populations diverged across riverine barriers (Ayres & Clutton‐Brock, 1992; Bates, 1863; Hershkovitz, 1977; Mayr, 1942; Sick, 1967; Wallace, 1853); the “refuge hypothesis” in which forests fragmented during the cold, dry Pleistocene glaciation cycles, causing isolation and divergence in small forest patches (Haffer, 1969, 1974, 1982; Prance, 1982; Vanzolini, 1973; Vanzolini & Williams, 1970); and an amalgamate “river‐refuge hypothesis,” in which speciation was promoted by a combination of river barriers and climate‐driven vegetation changes (Ayres & Clutton‐Brock, 1992; Haffer, 1992, 1993). These hypotheses have been widely employed as the context for studies of Neotropical biodiversity and the mechanisms of its production (e.g., Gascon et al., 2000; Haffer, 2008; Patton & Silva, 2005; Richardson et al., 2001; Weir, 2006). However, because the early scientific focus was primarily on the Amazon (Amorim, 1991; Cracraft, 1985; DeMenocal, 2004; Haffer, 1969, 1997; Plana, 2004; but see Fjeldså, 1994; Mayr & O'Hara, 1986) and given political instability in tropical Africa (Greenbaum, 2017; Siddig, 2019; Tolley et al., 2016), rigorous testing of the predictions stemming from these hypotheses has been neglected for the West and Central African rainforests until only recently.

**FIGURE 1:**
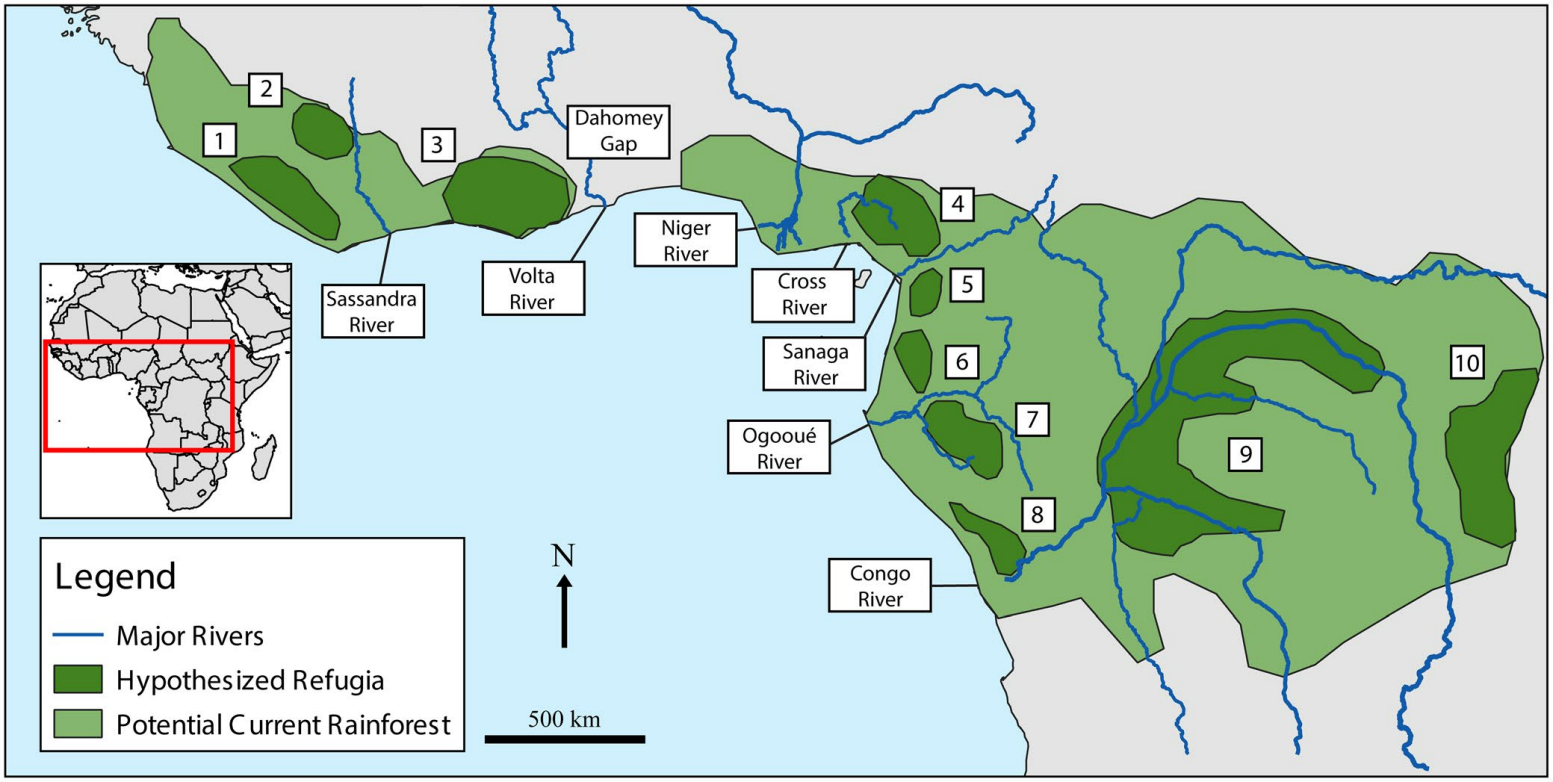
Locations of major rivers and hypothesized refugia (labeled 1–10) in West and Central Africa, adapted from Maley (1996)

Based on pollen core records (Bonnefille & Riollet, 1988; Brenac, 1988; Girese et al., 1994; Maley, 1987, 1989, 1991; Maley & Brénac, 1987; Maley & Livingstone, 1983; Sowunmi, 1991) and species distribution data (Colyn, 1987, 1991; Richards, 1963; Rietkerk et al., 1995; Sosef, 1991), Maley (1996) proposed several Pleistocene rainforest refugia for sub‐Saharan Africa that are still considered today (e.g., Bell et al., 2017; Hughes et al., 2017; Huntley et al., 2019; Jongsma et al., 2018; Larson et al., 2016; Penner et al., 2011; Portik et al., 2017; Figure 1). Many of these hypothesized refugia are located in highland areas (e.g., the Cameroon Volcanic Line and the Albertine Rift; Figure 1, refugia 4 and 10, respectively). However, a major fluvial refuge, that is, a refugium associated with a river, located in the gallery forests around the Congo River (Figure 1, refugium 9), has been supported by pollen core data (Maley, 1996), and distributional patterns of multiple bird (Huntley et al., 2018; Levinsky et al., 2013), mammal (Colyn et al., 1991; Levinsky et al., 2013), and plant taxa (Robbrecht, 1996).

Major river barriers in West and Central Africa include the Volta, Sanaga, Ogooué, Congo, Niger, and Cross Rivers (Figure 1). The exact ages of many of these rivers are unknown but are generally estimated to date back to the Late Mesozoic to Early Cenozoic (80–35 mya; Couvreur et al., 2021; Goudie, 2005; Stankiewicz & de Wit, 2006). However, while the Congo basin is quite old (Flügel et al., 2015; Stankiewicz & de Wit, 2006), the present course of the Congo River appears to be much younger, dating to the mid to late Miocene and corresponding to the uplift of the East African Rift (Flügel et al., 2015; Stankiewicz & de Wit, 2006; Takemoto et al., 2015).

Numerous phylogeographic studies have supported the importance of rivers, refugia, or both as drivers of diversification across disparate plant and animal species. Rivers alone have been shown to be important barriers for some species of primates (Mitchell et al., 2015; Telfer et al., 2003), shrews (Jacquet et al., 2015), and frogs (Charles et al., 2018; Penner et al., 2011, 2019; Wieczorek et al., 2000; Zimkus et al., 2010), but do not appear to represent an important barrier for many plant species (Dauby et al., 2014; Debout et al., 2011; Hardy et al., 2013; Ley et al., 2014; Lowe et al., 2010). Refugia are suggested to have played an important role in the diversification of rodents (Bohoussou et al., 2015; Nicolas et al., 2011, 2012), primates (Clifford et al., 2004; Haus et al., 2013; Tosi, 2008), frogs (Bell et al., 2017; Jongsma et al., 2018), lizards (Allen et al., 2019; Leaché et al., 2017), birds (Fjeldså & Bowie, 2008), pangolins (Gaubert et al., 2016), and rainforest plants (Born et al., 2011; Budde et al., 2013; Daïnou et al., 2010; Dauby et al., 2010; Duminil et al., 2015; Faye et al., 2016; Gomez et al., 2009; Hardy et al., 2013; Ley et al., 2014, 2016; Lowe et al., 2010). In some cases, divergence patterns match both refugial and riverine predictions (Anthony et al., 2007; Barej et al., 2011; Bohoussou et al., 2015; Gonder et al., 2011; Jacquet et al., 2014; Jongsma et al., 2018; Leaché & Fujita, 2010; Leaché et al., 2019; Marks, 2010; Portik et al., 2017), suggesting that both may have played roles simultaneously—or in combination—in evolutionary diversification. However, because of the spatial overlap of refugia with montane and riverine systems (Hofer et al., 1999, 2000), and the sparse pollen core and fossil records for the tropics (Colinvaux et al., 1996; Maley & Brenac, 1998), distinguishing between these three hypotheses has been difficult, especially when relying on phylogeographic data alone.

The three major allopatric diversification hypotheses make the following predictions regarding species diversification patterns in tropical African forests (a) river hypothesis: boundaries between population distributions should correspond to riverine barriers and the ages of populations should be relatively old, corresponding to the ages of the rivers; (b) refuge hypothesis: population distributions should be concordant with locations of hypothesized rainforest refugia during cold, dry periods, and populations are predicted to be relatively young, corresponding to the Pleistocene glaciation cycles; (c) river‐refugia hypothesis: population distributions should be correlated with the locations of rainforest refugia and bounded by rivers barriers, or will have been confined to refugial locations and additionally subdivided by rivers. Under this scenario, the timing of population splits should correspond to ages of rivers but would be expected to show patterns of range expansion and contraction for niche model projections to the Pleistocene.

**FIGURE 2:**
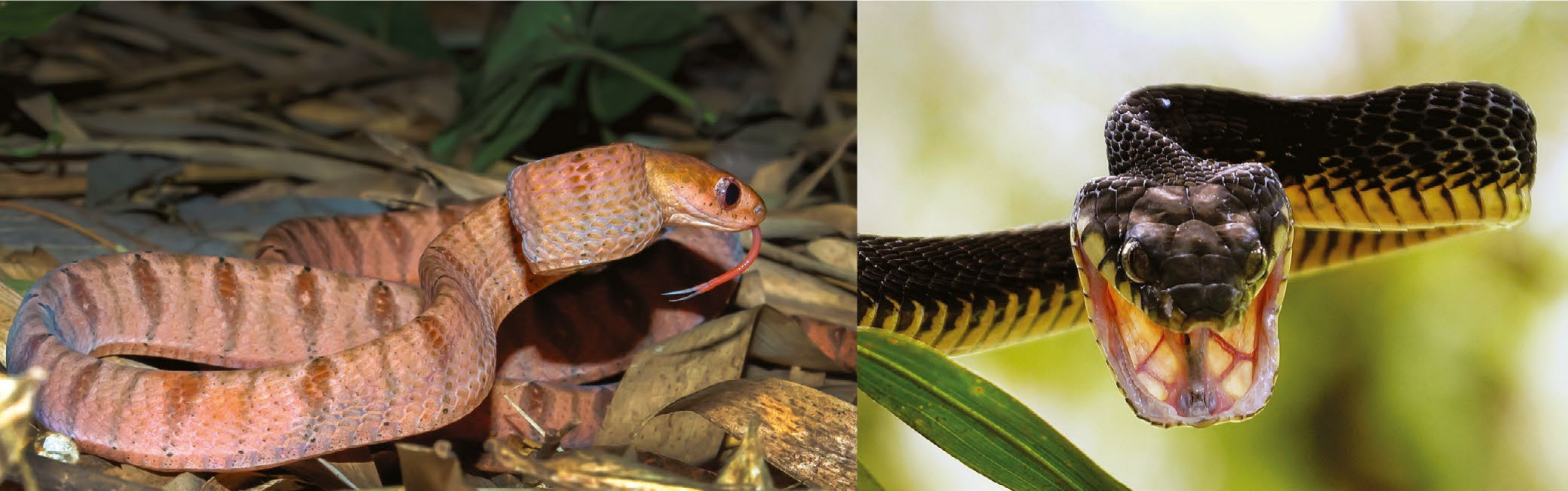
Left: *Toxicodryas pulverulenta*. Right: *Toxicodryas blandingii* (male). Both photographs were taken in Banalia, Tshopo Province, DR Congo. Photograph credits Konrad Mebert

In this study, we use the snake genus *Toxicodryas* as a model system to test multiple predictions derived from these hypotheses. The genus *Toxicodryas* consists of two species of large‐bodied, rear‐fanged, venomous sub‐Saharan African snakes, *T. blandingii,* and *T. pulverulenta* (Figure 2). For most of the 20th century, these taxa were placed in the Asian genus *Boiga* (Schmidt, 1923), and some authors still classify them as such, but recent phylogenetic analyses recover them as the sister genus to the African egg‐eating snakes, *Dasypeltis* (Pyron et al., 2013; Weinell et al., 2020). No in‐depth phylogenetic or phylogeographic analysis has been done within *Toxicodryas*. Both known species are primarily arboreal, feeding mainly on birds, bats, frogs, and arboreal lizards (Akani et al., 1998; Chippaux & Jackson, 2019; Nagy et al., 2011; Spawls et al., 2018). Because of their general arboreality, these species are predicted to have distributions strongly correlated with forest and woodland habitats. In addition, *Toxicodryas* is widely distributed within the Congo Basin and broadly across West and Central Africa, making this genus a suitable system for testing the competing predictions of the river, refugia, and river‐refugia hypotheses.

Recent advances in paleo‐climate modeling and genome‐scale DNA sequencing have opened new avenues to testing classic hypotheses of tropical rainforest speciation (Bell et al., 2017; Leaché et al., 2019; Portik et al., 2017). In this study, we integrate dated phylogeographic inference, population structure analyses, and machine learning‐based demographic modeling to identify the timing of divergence as well as the location and permeability of past and present dispersal barriers. These genetic data are combined with paleo‐distribution and climate stability modeling to determine the congruence of historical distributions with the refugial and river‐refugial hypotheses. Our results demonstrate that, although population distributions alone could be congruent with any of the three hypotheses, diversification times predate the Pleistocene, a finding that aligns with predictions of the river‐barrier hypothesis. Moreover, historical demographic analyses support models of no migration among populations since the time of divergence, and no signatures of population bottleneck and subsequent expansion were identified, as predicted under the refuge hypothesis. Additionally, species paleo‐distribution and climate stability modeling show no suggestion of suitable habitat contraction during or since the Pleistocene. Together, our analyses allow us to reject predictions of refugia hypotheses in favor of the prevailing role of riverine barriers in shaping, structuring, and maintaining diversity in this generally arboreal, forest‐associated group of endemic African snakes.

## 2 MATERIALS AND METHODS

### 2.1 Sampling

We obtained 20 specimens of *Toxicodryas* (seven *T. blandingii* and 13 *T. pulverulenta*) through fieldwork and from various museums (see Table S1). Sampling was representative of the known range of each species throughout the upper and lower Guinean forest blocks of West and Central Africa including the countries of Guinea, Liberia, Ghana, Cameroon, Gabon, and Democratic Republic of the Congo (DRC). Museum catalog numbers, GenBank accession numbers, and locality data for each specimen are presented in Table S1.

### 2.2 Genetic data collection, bioinformatic processing, and locus assembly

Tissue samples were preserved in 95% ethanol or RNAlater™ (Sigma‐Aldrich), and genomic DNA (gDNA) was extracted using the Maxwell RSC system (Promega). The nuclear gene c‐mos and the mitochondrial gene cytochrome *b* (cyt *b*) were PCR‐amplified for each individual using standard primers (c‐mos: S67, S68; Lawson et al., 2005; cyt *b*: L4910B, H15720; Burbrink et al., 2000) and sequenced on an ABI 3730 capillary electrophoresis system (Applied BioSciences^®^). Electropherograms were edited manually in Geneious v5.6.7 (http://www.geneious.com, Kearse et al., 2012), and resulting sequences were aligned in MAFFT v.5 with default parameters (Katoh & Kuma, 2002).

We also sequenced genome‐wide anonymous nuclear markers for each individual following a modified version of the ddRADseq protocol of Peterson et al., (2012). For each individual, a total of 300–500 ng of gDNA were double digested using the restriction enzymes *Sbf*I (restriction site 5′‐CCTGCAGG‐3′) and *Msp*I (restriction site 5′‐CCGG‐3′). The resulting double digestion products were then bead‐cleaned with AmpureXP beads (Agencourt) and individually barcoded using custom oligonucleotide adapters. Pooled samples were size‐selected to a mean insert length of 541 base pairs (bp) (487–595 bp range) with internal standards with a Pippin Prep™ (Sage Science, Beverly, MA, USA). Resulting postligation products were amplified for eight cycles with a high‐fidelity polymerase (Phusion™, New England Biolabs). An Agilent TapeStation was used to determine the final fragment size distribution and concentration of each pool. Library pools were combined in equimolar amounts for sequencing on one Illumina HiSeqX lane (with a 10% *Phi*X spike‐in and 150 bp paired‐end reads).

Illumina reads from the ddRAD libraries were processed using STACKS v. 2.4 (Catchen et al., 2013). Because the ddRAD protocol generates strand‐specific libraries, prior to read filtering and assembly, we used a read‐stitching approach (Hime et al., 2019) to join the first read from an Illumina read pair with the reverse complement of the second, recapitulating the original orientation of fragments in the genome. Stitched reads were quality‐filtered and demultiplexed by individual with the process_radtags function in STACKS with the following parameters: demultiplex each library by in‐line barcode, check for both restriction enzyme cut sites, remove any read with an uncalled base, rescue barcodes and RAD‐Tags, and discard any read with average Phred quality score <20 over sliding windows of 15% of the total read length. Next, we used STACKS to de novo assemble filtered and stitched Illumina read pairs.

We aimed to produce three separate ddRAD data sets, including one for *T. blandingii*, one for *T. pulverulenta*, and a combined data set comprising both species. Because the optimal de novo assembly of ddRADseq data can vary widely across taxa (Paris et al., 2017; Shafer et al., 2017), we tested a range of assembly parameters to optimize the recovery of putatively single‐copy orthologous loci. Final assembly parameters were selected based on the methods of Paris et al. (2017). According to their recommendations, in USTACKS, we kept m (the minimum number of reads needed to form a stack) at 3 while in CSTACKS, we varied *M* (the number of mismatches allowed during loci formation) and *n* (the number of mismatches allowed during catalog formation) until we identified the parameters at which the maximum number of polymorphic loci were available across 80% (*r* = 0.8) of the population. For our data, this was found to be *M* = 5 and *n* = 15. Further parameters were tested in POPULATIONS separately for each species and for the genus as a whole in order to balance missing data and number of polymorphic loci. Within *T. blandingii* and *T. pulverulenta*, the percent missing data were low (5% and 7.3% missing data, respectively) and no further processing was needed, and *r* = 0.8 was used. Because of dissimilarity between the two species causing high levels of missing data in the combined data set, further restrictions were implemented. For the genus‐wide data set, we set *r* = 0.5 and *p* = 4 [p is the minimum number of populations in which a locus must be present (here 4/5)]. This approach increased the number of informative loci, but also the amount of missing data. For each of our three separate data sets, we generated a data set comprising only a single random SNP per locus (for population clustering analyses and demographic modeling), and another data set comprising full‐length sequences for all loci (for use in phylogenetic reconstruction).

### 2.3 Phylogenetic analyses

We concatenated our Sanger data set (c‐mos and cyt *b*) and implemented bModelTest in Beast v. 2.6.2 to assess all possible substitution models for each gene using a Bayesian approach (Bouckaert & Drummond, 2017). We conducted a time‐calibrated analysis on our partitioned data set in Beast v. 2.6.2 (Bouckaert et al., 2019) using a relaxed log‐normal clock and a Yule tree prior assuming a constant lineage birth rate. Dating analyses were based on three fossils for calibration in the outgroup, one at the Elapoidea + Colubridae node (minimum age: 30.9 Mya), one at the *Heterodon* + *Farancia* node (minimum age: 12.08 Mya), and one at the *Naja* + *Hemachatus* node (minimum age: 17.0 Mya). Fossil ages and placement were based on Head et al. (2016). Two runs of 100,000,000 generations were conducted and logged every 10,000 generations. Convergence was assessed using Tracer v. 1.7 (Rambaut et al., 2018). A burn‐in of 10% was used to create a maximum clade credibility tree. Node ages are based on median tree heights.

We analyzed our SNP data set, including all samples of both species of *Toxicodryas*, using both species tree summary quartet and maximum likelihood phylogenetic methods. The quartet method was implemented through SVDquartets (Chifman & Kubatko, 2014) in PAUP* v. 4.1a166 (Swofford, 2003). We sampled all possible quartets and assessed support using 100 nonparametric bootstraps, and species tree topology was summarized with DendroPy v. 4.4.0 (Sukumaran & Holder, 2010). We ran a maximum likelihood analysis of our genus‐wide SNP data set in IQtree v. 1.6.12 (Nguyen et al., 2014) using 10,000 ultrafast bootstraps (Hoang et al., 2018) and the ModelFinder function to choose the best substitution model (Kalyaanamoorthy et al., 2017). As no outgroups were included in our SNP data set, for both SVDquartets and IQtree the placement of the root of each phylogeny was chosen to match that of the Sanger phylogeny.

### 2.4 Assessing genetic structure

We used multivariate, Bayesian, and admixture‐based analyses to assess population structure. In all analyses, clustering algorithms were run on three data sets separately for comparison (*T. blandingii*, *T. pulverulenta*, and both species combined [genus *Toxicodryas*]). A discriminant analysis of principal components (DAPC) was run using Adegenet v. 2.1.1 (Jombart & Ahmed, 2011). This approach uses discriminant functions to maximize variation among clusters and minimize variation within clusters. The best‐clustering scheme was chosen based on Bayesian information criterion (BIC) scores. Numbers of clusters (K) ranging from 1–10 for the genus‐level analysis and 1–5 for each species were evaluated and a discriminant function analysis of principal components (DAPC) was performed based on the number of suggested clusters. Ancestry proportions of all individuals were inferred using LEA v. 1.6.0 (Frichot & François, 2015) through the Bioconductor v. 3.4 package. The sNMF function was used to assess K values from 1–10 for the genus‐level analysis and 1–5 for each species with 20 replicates each. Individual admixture coefficients were estimated and the value of K that minimized cross‐entropy was selected (François, 2016; Frichot et al., 2014). Population structure and admixture were also tested using the Bayesian method STRUCTURE v. 2.3.4 (Falush et al., 2003; Pritchard et al., 2000). Each data set was evaluated for K = 1–10 for the genus‐level analysis and 1–5 for each species, with 10 runs per K and a MCMC burn‐in of 10,000 steps followed by 100,000 steps (Porras‐Hurtado et al., 2013). Results were evaluated using the Evanno method (Evanno et al., 2005) and plotted through the R package pophelper v. 2.3.0 (Francis, 2017).

### 2.5 Demographic modeling and analysis of gene flow

To test for historic and recent gene flow between our populations, we used the R package delimitR (Smith & Carstens, 2020; https://github.com/meganlsmith/delimitR). This program uses a binned multidimensional folded site frequency spectrum (bSFS; Smith et al., 2017) and a random forest machine learning algorithm to compare speciation models such as no divergence, divergence with and without gene flow, and divergence with secondary contact (Smith & Carstens, 2020). A bSFS was used because it stores the observed frequencies of the minor alleles for multiple populations and bins them to avoid inference problems associated with sampling too few segregating sites (Smith et al., 2017; Terhorst & Song, 2015). Demographic histories are simulated using the multi‐species coalescent model implemented through fastsimcoal2 (Excoffier et al., 2013) under a user‐specified guide tree and set of priors on divergence times, population sizes, and migration rates. The random forest classifier then creates a user‐defined number of decision trees from a subset of the prior. Each decision tree compares the empirical bSFS to the SFS of each simulated speciation model and votes for the most similar model. The demographic model with the largest number of votes is chosen as the best model. Out‐of‐bag error rates are used to assess the power of the random forest classifier. The posterior probability of the selected model is then calculated by regressing against the out‐of‐bag error rates following Pudlo et al. (2015).

We created folded multidimensional site frequency spectrums for the two *T. blandingii* clades and the two Central African *T. pulverulenta* clades using easySFS (https://github.com/isaacovercast/easySFS), a wrapper for ∂a∂i (Gutenkunst et al., 2009). The West African *T. pulverulenta* clade was not included because of the low sample size available for this lineage. We simulated 100,000 data sets under four models: no divergence (Model 1), divergence without gene flow (Model 2), divergence with secondary contact (Model 3), and divergence with gene flow (Model 4). Priors for both models were drawn from uniform distributions for population size: 10,000–1,000,000 haploid individuals (twice the number of estimated diploid individuals), divergence time: 20,000–2,000,000 generations, migration rate: 0.000005–0.005 corresponding to 0.05–5 migrants per generation. We then coarsened our empirical site frequency spectra to 10 bins each. Our out‐of‐bag error rates were calculated, and 500 random forest classifiers were simulated using 100,000 pseudo‐observed data sets for each model. A confusion matrix was calculated to determine how often the correct model was selected and posterior probability for the “best” model was estimated for each species.

The R package rangeExpansion (Peter & Slatkin, 2013, 2015) was used to assess signatures of population size change in the two *T. blandingii* clades and the two Central African *T. pulverulenta* clades. The West African *T. pulverulenta* clade was excluded because of its small sample size. This program implements a founder effect algorithm using a steppingstone model, assuming that each colonizing event is associated with a founder event, to determine whether a population shows signatures of expansion or equilibrium isolation‐by‐distance (Peter & Slatkin, 2013, 2015). If population expansion is identified, the program will infer the strength of the founder effect and the most likely expansion origin (Peter & Slatkin, 2013, 2015).

### 2.6 Species delimitation

We conducted a species delimitation analysis on our Sanger data set using a Bayesian approach through BPP v.4.2.9 (Flouri et al., 2018; Yang, 2015) and a user‐specified guide tree (Rannala & Yang, 2013; Yang & Rannala, 2010). Following Yang and Flouri (2020), we used the default prior for theta (θ = 0.002) and calculated the tau prior based on the estimated divergence times (τ = 0.036). We used a burn‐in of 20,000, a sampling size of 200,000, and a sampling frequency of two. The analysis was run twice with a random seed to ensure consistency.

Additionally, the package DelimitR (Smith & Carstens, 2020; https://github.com/meganlsmith/delimitR) was used to conduct a species delimitation analysis on our ddRAD data set. As described above for the demographic analysis, this program uses a binned multidimensional folded site frequency spectrum (bSFS; Smith et al., 2017) and a random forest machine learning algorithm to compare the speciation models: no divergence, divergence with gene flow, divergence with secondary contact, and divergence without gene flow (Smith & Carstens, 2020). Each scenario is simulated using a multi‐species coalescent model implemented through fastsimcoal2 (Excoffier et al., 2013) and user‐specified priors on divergence times, population sizes, and migration rates. The empirical bSFS to the SFS of each simulated speciation model is then compared by the random forest classifier and posterior probabilities, and out‐of‐bag error rates are calculated.

### 2.7 Species distribution modeling

Occurrence data for each species were obtained from the specimens used in this study, “expert” identified individual occurrences from the Global Biodiversity Information Facility (GBIF), and research‐grade locality records from iNaturalist (www.inaturalist.org) that could be visually identified by the authors. Duplicate records were removed, and points were thinned within a distance of 10 kilometers using the spThin package (Aiello‐Lammens et al., 2015) in R v. 3.4.4 (R Core Team, 2019). This resulted in a total of 43 *T. blandingii* localities and 30 *T. pulverulenta* localities (Figure S1). A subset of points from each data set was set aside for model calibration (75%) and internal testing (25%) following Cobos et al. (2019).

Environmental data were obtained from the WorldClim database v. 1.4 (Hijmans et al., 2005). Fifteen of the 19 bioclim variables were downloaded at a 2.5‐min resolution. We excluded bio8 (Mean Temperature of Wettest Quarter), bio9 (Mean Temperature of Driest Quarter), bio18 (Precipitation of Warmest Quarter), and bio19 (Precipitation of Coldest Quarter) which, as combinations of other variables, are known to create artifacts in distribution models (Escobar et al., 2014). The same 15 variables were used for the Last Glacial Maximum (LGM) under three general circulation models (GCMs): CCSM4, MIROC‐ESM, and MPI‐ESM‐P. In order to reduce spatial autocorrelation, principal component analyses (PCAs) were performed on present bioclim variables and projected to the LGM for the extent of sub‐Saharan Africa.

Model calibration areas were defined as a 1,000‐km buffer around occurrence points for each species. Model calibration, creation, projection, and evaluation were done using the R package kuenm (Cobos et al., 2019). In order to calibrate our models, we created 1,479 candidate models for each species by combining three sets of environmental predictors (PCAs 1–6, 1–5, 1–4), 17 possible regularization multipliers (0.1–1.0 at intervals of 0.1, 2–6 at intervals of 1, and 8 and 10), and all combinations of five feature classes (linear = l, quadratic = q, product = p, threshold = t, and hinge = h; Cobos et al., 2019).

Candidate models were run in Maxent (Phillips et al., 2006) and chosen based on significant partial receiver operating characteristic (ROC) scores (Peterson et al., 2008), omission rates of E ≤ 5% (Anderson et al., 2003), and corrected Akaike Information Criterion AICc ≤ 2 to minimize model complexity (Warren & Seifert, 2011). These models determined the parameter set used for final model creation.

Final models were created for each species using the full set of occurrence records and the parameters chosen during model calibration. Models were run in Maxent with ten bootstrap replicates and logistic outputs. After models were run in the present, they were projected to the LGM and mid‐Holocene for the three GCMs. The mobility‐oriented parity (MOP) index was used to test for model extrapolation (Soberón & Peterson, 2005). Models were visualized in QGIS 3.4 and thresholded to 5% to create presence‐absence maps. Models from each time period were summed to estimate potential LGM and mid‐Holocene distributions as well as continuous stability maps (Devitt et al., 2013; Yannic et al., 2014).

## 3 RESULTS

### 3.1 Genetic data collection, bioinformatic processing, and locus assembly

Our concatenated c‐mos and cyt *b* data set (Sanger data set hereafter) consisted of 1,237 bp, including indels. Both genes were represented in all samples with the exception of c‐mos for the outgroup *Contia longicaudae*. Information on samples used in the Sanger analysis, including museum catalog number and GenBank accession number, can be found in the Supporting Information. After filtering (see Section 2, above), our genus‐level ddRAD data set consisted of 2,848 loci with 20.7% missing data (here defined as proportion of missing loci across all individuals), and an effective mean per‐sample depth of coverage of 78.7x ± 13.6x. Our *T. blandingii* data set consisted of 7,231 loci with 5.0% missing data, and an effective mean per‐sample depth of coverage of 83.6x ± 12.0x. Our *T. pulverulenta* data set consisted of 4,471 loci with 7.3% missing data, and an effective per‐sample mean depth of coverage of 77.9x ± 14.6x. The concatenated ddRAD data set used for phylogenetic analyses had a length of 450,512 bp and 3,024 SNPs.

### 3.2 Phylogenetic structure and divergence dating

**FIGURE 3:**
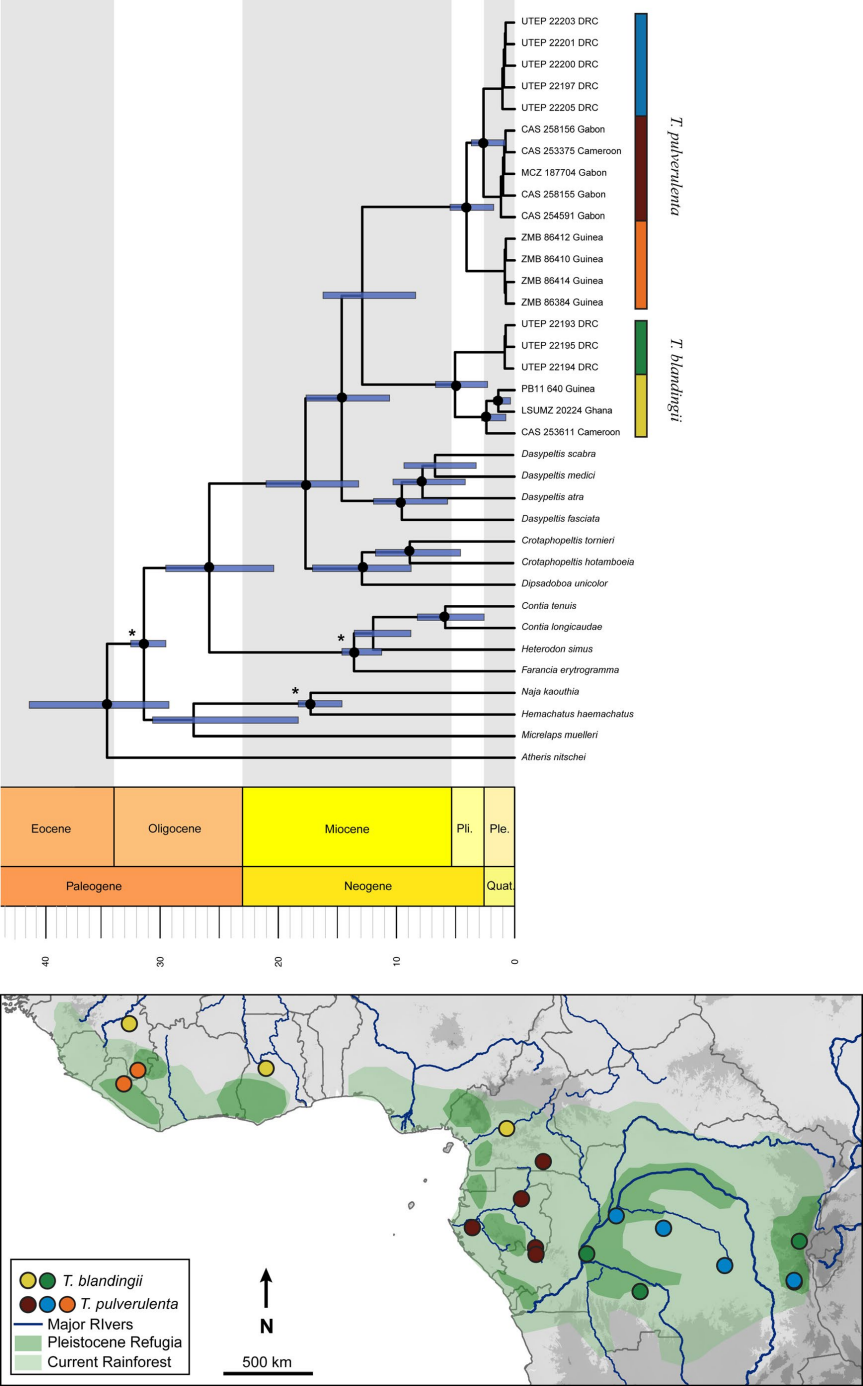
Top: A time‐calibrated Bayesian phylogeny for *Toxicodryas* with c‐mos and cyt *b* genes. Highly supported nodes (PP ≥ 0.9) are denoted with a black circle. Fossil‐calibrated nodes are denoted with an asterisk. Node bars represent 95% confidence intervals. RADseq phylogenies showed identical topologies. Bottom: *Toxicodryas* clade distributions overlaid onto a map of elevation, major rivers and hypothesized rainforest refugia

Broad‐scale phylogenetic relationships estimated in analyses of our Sanger and SNP data sets were identical in topology, with strongly supported internal nodes throughout (Figure 3; Figure S2). Our two‐locus Sanger tree and our 2,848‐locus ddRAD SNP trees both supported two divergent lineages of *T. blandingii*, in West and Central Africa, respectively, and three divergent lineages of *T*. *pulverulenta*, one from West Africa and two in Central Africa, north and south of the Congo River (Figure 3). Fossil‐calibrated divergence dating suggests that *T. blandingii* and *T. pulverulenta* diverged in the mid‐Miocene (median age 12.2 Mya). Diversification within each species is estimated to have taken place primarily in the Pliocene, with the two clades in *T. blandingii* diverging around 4.3 Mya, the West African clade of *T. pulverulenta* diverging around 3.3 Mya, and the two Central African clades diverging around 1.9 Mya (Figure 3).

### 3.3 Population structure

**FIGURE 4:**
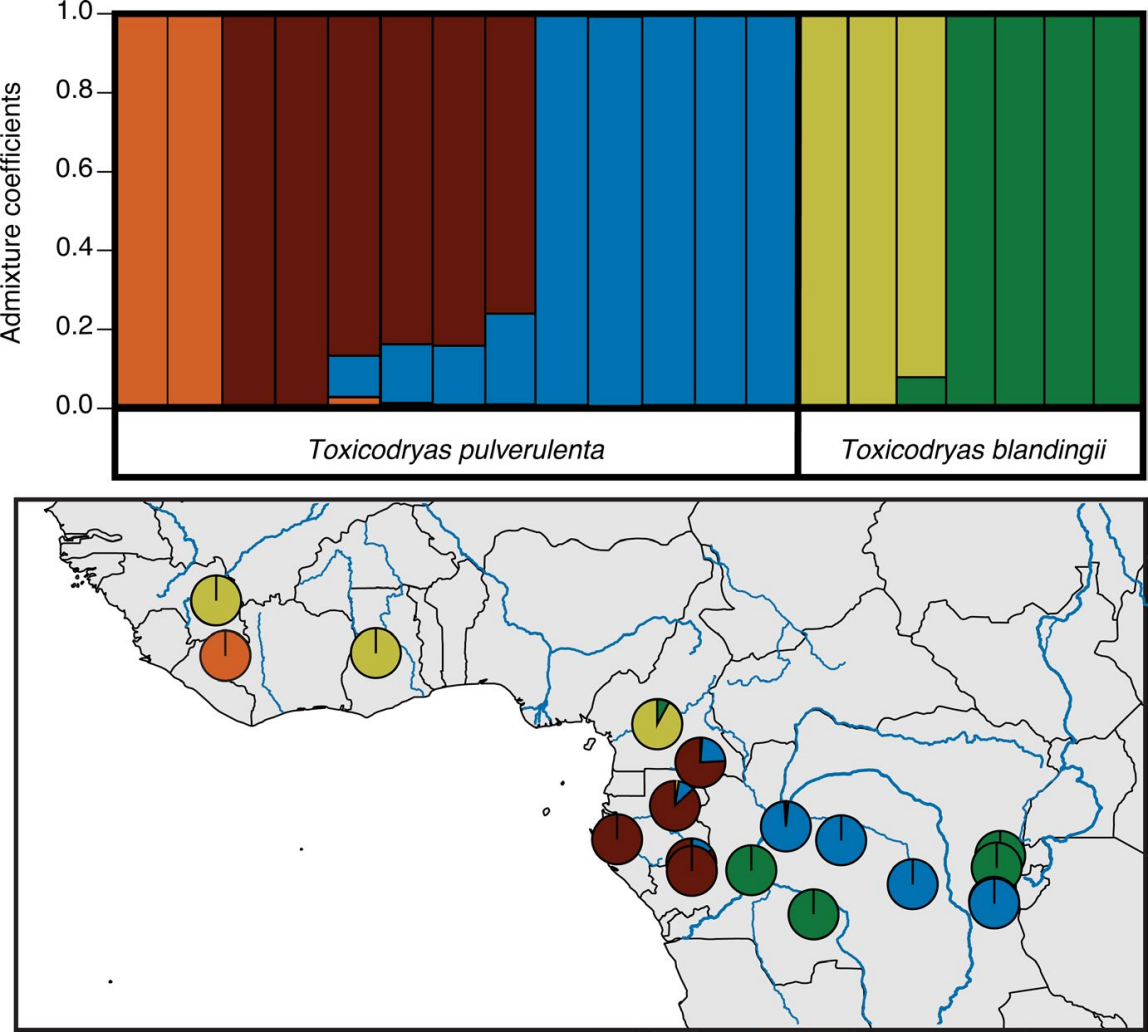
Population structure of the genus *Toxicodryas*. Top: Bar plot of population structure and membership probabilities for K = 5 analyzed in LEA. Bottom: Geographic representation of population structure for K = 5 overlaid onto a map of major rivers

A comparison of BIC values from the genus‐level DAPC analyses suggested a total of five genetic clusters, with two populations in *T. blandingii* and three in *T. pulverulenta*, matching the clades identified in the phylogenetic analyses (Figure S3). Our admixture‐based method, LEA, identified two distinct genetic clusters at the genus level, corresponding to the two *Toxicodryas* species, and the same two populations for *T. blandingii* and three populations for *T. pulverulenta* as suggested by DAPC (Figure 4). A low amount of admixture was identified in the Cameroonian sample of *T. blandingii*, and varying levels of admixture were suggested for the Gabonese samples of *T. pulverulenta* (Figure 4). The population assignment of individuals between the two clustering methods was identical; however, admixture between populations was not detected by DAPC. Similarly, STRUCTURE suggested two populations at the genus level, and two in *T. blandingii*, but combined the Central African clades and suggested two populations, instead of three, for *T. pulverulenta*. Three populations were supported as the second highest ΔK and showed identical admixture proportions to those from LEA. We used five populations for our remaining analyses because multivariate‐based analyses such as LEA and DAPC do not make assumptions about Hardy–Weinberg equilibrium and may be preferable over Bayesian methods such as STRUCTURE when sample sizes are small or uneven (Puechmaille, 2016).

### 3.4 Demographic modeling and analysis of gene flow

**FIGURE 5:**
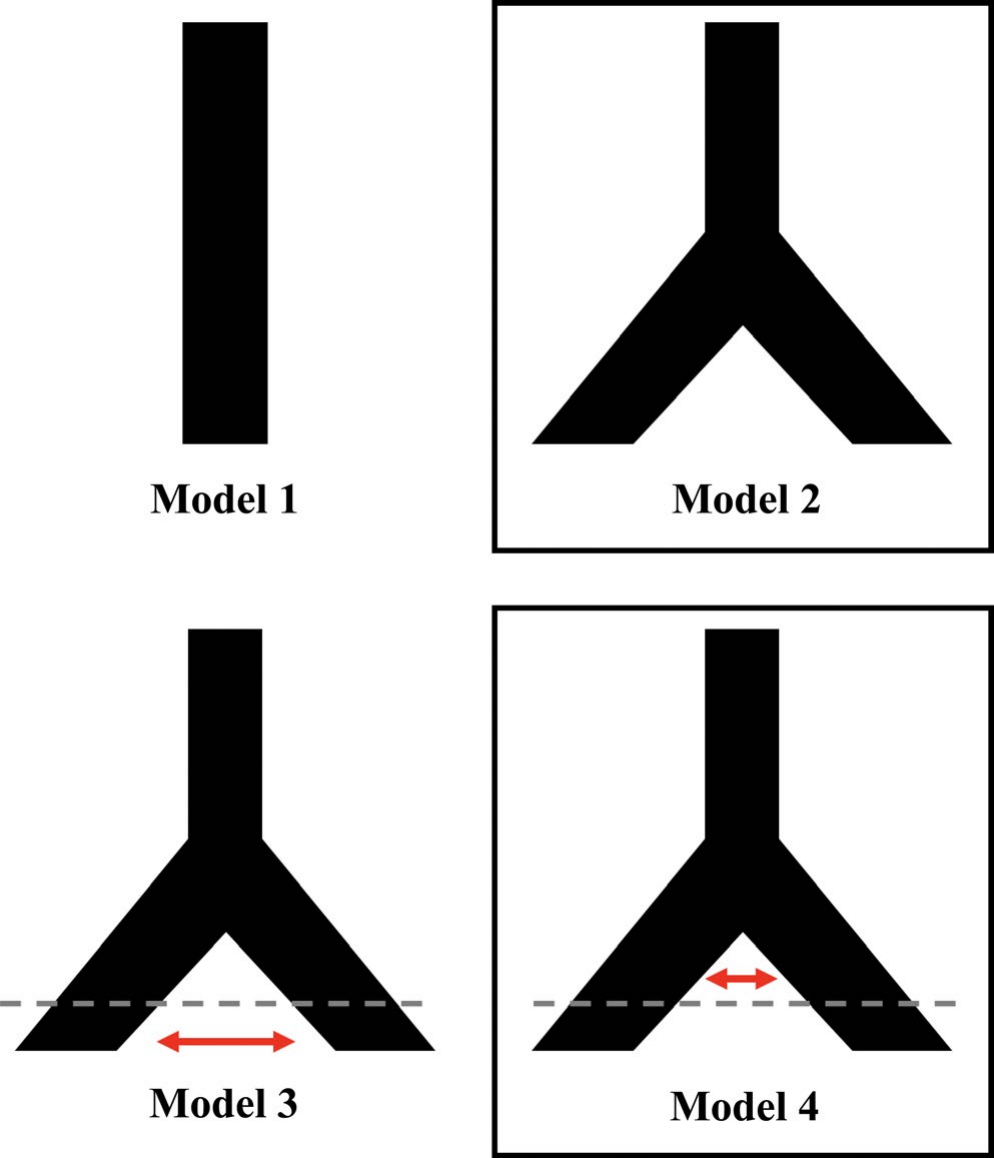
Four demographic models tested using DelimitR. Model 1: no divergence, Model 2: divergence without gene flow, Model 3: divergence with secondary contact, and Model 4: divergence with gene flow. Model 2 was chosen for *Toxicodryas blandingii*, and Model 4 was chosen for the two Central African clades of *T. pulverulenta*

Using machine learning‐based demographic model selection, we identified divergence without gene flow as the best model for *T. blandingii* with a posterior probability of 0.68, and divergence with gene flow for *T. pulverulenta* with a posterior probability of 0.63 (Figure 5). For both species, models representing no divergence and divergence with secondary contact received very low support (Tables S2 and S3). The out‐of‐bag error rate for *T. blandingii* was 17.3% and for *T. pulverulenta* was 22.8%, with all of the misclassifications being between highly similar models (i.e., between divergence with or without gene flow, as opposed to between divergence and no divergence). While it is possible that small sample sizes for several of our populations may have negatively impacted the power of our demographic analyses, our values for posterior probability and out‐of‐bag error rate are similar to those obtained by Smith and Carstens (2020), suggesting that the impact was minimal. The confusion matrix and number of votes per model can be found in Tables S2 and S3.

Population size analyses for demographic signatures of range expansion versus equilibrium isolation‐by‐distance strongly rejected the expansion model for each of the four populations tested (West African *T. blandingii*: *p* = 6.38; Central African *T. blandingii*: *p* = 28.01; Gabon *T. pulverulenta*: *p* = 833.43*;* Congo Basin *T. pulverulenta*: *p* = 0.10). Accordingly, the strength of the founder effect (q) for each population was generally small, and the founder distance (d) was large (West African *T. blandingii*: q = 0.000037, d = 137.46; Central African *T. blandingii*: q = 0.000028, d = 180.61; Gabon *T. pulverulenta:* q = 0.000042, d = 120.65*;* Congo Basin *T. pulverulenta*: q = 0.00017, d = 29.35).

### 3.5 Species delimitation

We tested five distinct clades of *Toxicodryas*: West African *T. blandingii*, Central African *T. blandingii*, West African *T. pulverulenta,* and two Central African clades of *T. pulverulenta* using the Bayesian species delimitation method BPP v. 4.2 (Flouri et al., 2018; Yang, 2015). Our analysis supported all five clades of *Toxicodryas* as distinct species with high posterior probability (pp = 0.98). Our analysis was run twice with random seeds to check for consistency, and both runs gave highly similar results. Similarly, DelimitR identified divergence without gene flow as the best model for *T. blandingii* and divergence with gene flow for *T. pulverulenta*. For both species, models suggesting lack of divergence or present‐day gene flow had very low probabilities (Tables S2 and S3).

### 3.6 Distribution modeling

**FIGURE 6:**
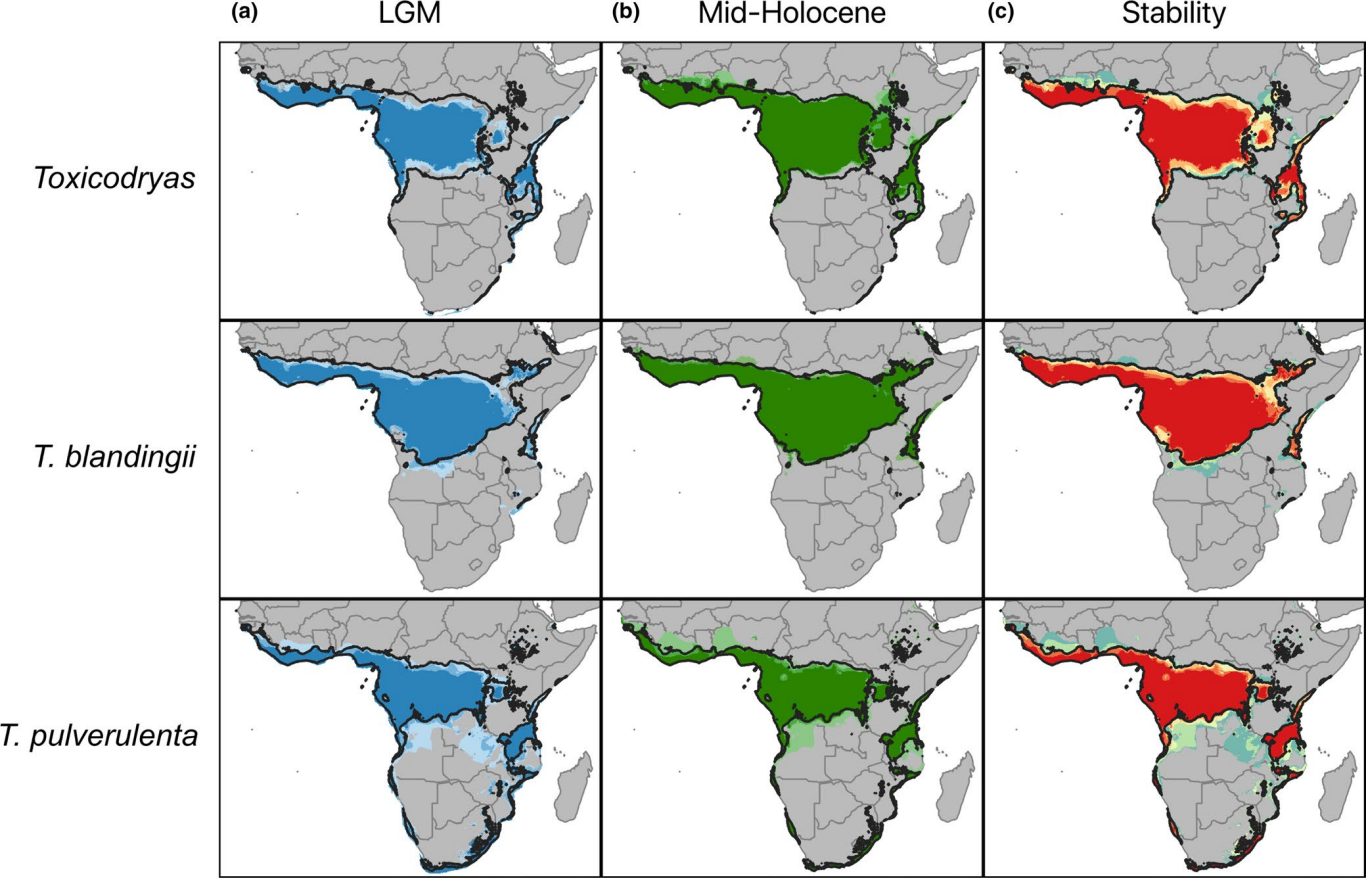
Paleo‐distribution models showing (a) the suitable habitat for *Toxicodryas* during the last glacial maximum (LGM). The shade of blue represents agreement between global climate models (GCMs) with the darkest blue indicating agreement between all three GCMs and the lightest blue indicating support from only one GCM. (b) The suitable habitat for *Toxicodryas* during the mid‐Holocene. The shade of green represents agreement between GCMs with the darkest green indicating agreement between all three GCMs and the lightest green indicating support from only one GCM. (c) The stability of suitable habitat across the LGM, mid‐Holocene, and present, with red indicating high stability and blue indicating low stability

Species distribution modeling suggested widely overlapping ranges for *T. blandingii* and *T. pulverulenta*, with both species documented from both rainforest and woodland habitats (Figure 6). Paleo‐distribution models for the LGM suggested a slight northern and southern contraction of suitable habitat for the genus in West and Central Africa. *Toxicodryas pulverulenta* showed evidence of a slight southward range expansion into Angola, while the range of *T. blandingii* remained stable (Figure 6a). The mid‐Holocene distribution was highly similar to the present‐day distribution for all data sets (Figure 6b).

Continuous climate stability maps estimating the areas of persistent suitable habitat from the LGM to the present suggest that the core distribution of each species has remained stable through time (Figure 6c). Instability in suitable habitat is only found on the edges of the species range, with the greatest potential for distribution change in southern Central Africa. No northward range expansion past the present day was estimated at any time scale in Central Africa, but lesser degrees of northward expansion may have been possible in West Africa.

## 4 DISCUSSION

The relative roles of rivers and refugia in shaping the high levels of species diversity in tropical rainforests have been widely debated for decades (e.g., Amorim, 1991; Colinvaux et al., 2001; DeMenocal, 2004; Haffer, 1969, 1997; Mayr & O'Hara, 1986; Vitorino et al., 2016; reviewed in Couvreur et al., 2021). Only recently has it become possible to take an integrative approach to answering these questions with genomic sequencing and paleo‐species distribution modeling (Leaché et al., 2019; Portik et al., 2017). Herein, we tested alternate predictions of the classic river, refuge, and river‐refuge hypotheses for terrestrial faunal diversification using a novel study system: the arboreal African snake genus *Toxicodryas*. We found strong support for predictions derived from the river hypothesis over the refuge and river‐refuge hypotheses, based on the ages, locations, and timing of gene flow between each of our populations, as well as a lack of support for suitable habitat and population size contraction during the last glacial maximum.

### 4.1 Species diversification

This study represents the first phylogenetic analysis of the genus *Toxicodryas*. Phylogenetic analyses of our two‐locus Sanger data set and 2,848‐locus RADseq SNP data set reveal two deeply divergent, strongly supported lineages in *T. blandingii* and three in *T. pulverulenta* (Figure 3; Figure S2). Although the two recognized species are broadly sympatric, clades within each species are generally situated allopatrically across river barriers. Of the three *T. pulverulenta* clades, one is distributed in West Africa (albeit with limited sampling) and two are distributed in Central Africa, separated by the western Congo River. The boundary between the two clades within *T. blandingii* is delimited somewhere between the Sanaga River in Cameroon and the Congo River in the DRC, and, while the species distribution reaches this area, no genetic sampling is available. Both rivers have frequently been interpreted as population barriers in other terrestrial vertebrates (Blackburn, 2008; Jongsma et al., 2018; Leaché & Fujita, 2010; Leaché et al., 2019; Portik et al., 2017), and a recent analysis of morphological data by Greenbaum et al. (in press) of all available *Toxicodryas* material, including voucher specimens of the genetic samples used herein, has identified the most likely biogeographic barriers within the genus to be the confluence of the Congo and Ubangi Rivers for the Central African populations of *T. blandingii* and *T. pulverulenta*, and the Niger Delta as the barrier for the West and Central African populations of *T. pulverulenta*.

Our population structure analyses are concordant with phylogenetic analyses supporting five distinct genetic clusters (Figure 4), and our species delimitation analyses suggest that all of these clusters represent distinct, independently evolving lineages. Minor levels of admixture seem to have occurred between the *T. pulverulenta* clades separated by the western Congo River, and between the two clades of *T. blandingii* in the sample collected at the Sanaga River (Figure 4); however, our demographic analyses do not support contemporary gene flow, suggesting that this admixture is a result of historic introgression or incomplete lineage sorting. In both species, the Congo River barrier seems to be stronger in the west where the river is wider, and the current is stronger. In eastern DRC, samples of clades from both species can be found on either side of this river (Figure 4).

### 4.2 Divergence time estimates

Divergence time estimates from a time‐calibrated phylogeny also support predictions derived from the river‐barrier hypothesis. *Toxicodryas blandingii* and *T. pulverulenta* diverged in the mid‐Miocene, and subsequent intraspecific diversification took place in the Pliocene (Figure 3). If the Pleistocene rainforest refugia hypothesis was supported, we would expect diversification times dating to the Pleistocene glaciation cycles, <2.5 million years ago. Similar mid‐Miocene and Pliocene divergence times have been noted for other widespread Central and West African taxa including frogs (Bell et al., 2017; Jongsma et al., 2018; Zimkus et al., 2017), and terrestrial snakes (Portillo et al., 2019), and similar West to Central African distribution splits have been seen in forest cobras (Wüster et al., 2018), frogs (Leaché et al., 2019), lizards (Allen et al., 2019), and shrews (Jacquet et al., 2015).

Greenbaum et al. (in press) identified two major river systems, the Congo/Ubangi and the Niger Delta, as likely barriers between *Toxicodryas* clades based on a combined molecular and morphological analysis of the genus. The Congo River and the Ubangi River, one of the Congo's tributaries, date back to the mid–late Miocene (Flügel et al., 2015; Stankiewicz & de Wit, 2006). The Congo is one of the largest rivers in the world, second only to the Amazon in discharge volume, and first in the world for depth. Much of its length, especially in the lower Congo, is characterized by swift‐flowing currents and waterfalls. The depth and intensity of this river have rendered it a well‐known barrier to many species including primates (Harcourt & Wood, 2012; Mitchell et al., 2015; Takemoto et al., 2015; Telfer et al., 2003), shrews (Jacquet et al., 2015), and other snakes (Portillo et al., 2019). The Niger is the third largest river in Africa and it likely originated approximately 29–34 million years ago (Chardon et al., 2016; Reijers, 2011), reaching its full extent by the mid‐Miocene as continental uplift progressed (Reijers, 2011). It is a biogeographic barrier for several species of frogs (Onadeko & Rödel, 2009; Rödel et al., 2014), primates (Eriksson et al., 2004), and shrews (Igbokwe et al., 2019).

### 4.3 Alternate biogeographic barriers

While the timing and locations of population divergences in this study correspond with river barriers, the Miocene was also a time of global climatic change characterized by dramatic cooling and vegetation shifts throughout sub‐Saharan Africa (Couvreur et al., 2021; Herbert et al., 2016; Jacobs, 2004; Menegon et al., 2014). Although most research surrounding the role of refugia in driving diversification has focused on the dramatic climate oscillations of the Pleistocene, it is likely that refugia are able to form during any period of climatic change (Haffer, 1997; Hampe & Jump, 2011; Jansson & Dynesius, 2002), but the role of possibly older refugia has received little attention in the literature (Couvreur et al., 2021; Hampe & Jump, 2011).

It is interesting to note that a number of biogeographic barriers that often play a role in species diversification in Central Africa do not appear to influence the genetic patterns seen in *Toxicodryas*. One of these biogeographic barriers is the Dahomey Gap, a dry forest‐savanna mosaic that separates the Upper and Lower Guinean forest blocks (Figure 1). Based on pollen cores and climatic modeling, the Dahomey Gap has existed in some form for at least the last 150,000 years (Allen et al., 2020; Dupont & Weinelt, 1996) and has played a variable role in species diversification in West Africa (Droissart et al., 2018; Leaché et al., 2020; Linder et al., 2012; Nicolas et al., 2010; Penner et al., 2011; White, 1979). The West African clade of *T. blandingii* crosses the Dahomey Gap and our species distribution analyses suggest that conditions are suitable for both species in that area, likely as a result of the forest‐mosaic providing suitable habitat (Chippaux & Jackson, 2019).

In addition, the two mountain ranges that bisect both species of *Toxicodryas*' ranges, the Cameroon Volcanic Line and the Albertine Rift, do not appear to impact the genetic structure of these species. Both mountain ranges originated approximately 30 million years ago with major geological developments occurring within the Miocene (Burke, 2001; Macgregor, 2015; Marzoli et al., 2000; Paul et al., 2014; Reusch et al., 2010) and both are continuing to undergo uplift and volcanism. Both are also biogeographically important in Central Africa as proposed Pleistocene refugia (Maley, 1996; Figure 1, refugia 4 and 10) and as barriers to dispersal for a variety of herpetofaunal species (e.g., Evans et al., 2011; Greenbaum et al., 2015; Menegon et al., 2014; Wüster et al., 2018; Zimkus & Gvoždík, 2013). However, they seem to have played neither role in the evolution of the genus *Toxicodryas*.

### 4.4 Population demography

We used machine learning‐based demographic model selection to test different gene flow scenarios between the populations in each of our two species. If river formation was the major driver of diversification in these species, we might expect to see divergence with low or no gene flow at the time of divergence as the river was forming (Figure 5: models 2 and 4), but we would be less likely to see recent gene flow. Alternatively, we might be more likely to observe recent gene flow if the Pleistocene refugia hypothesis was supported, as populations diverge and reunite during glaciation cycles (Smith & Carstens, 2020; Figure 5: model 3). Our demographic analyses indicate divergence without gene flow between the two *T. blandingii* clades and divergence with minor gene flow across the Congo River in the two Central African *T. pulverulenta* clades (Figure 5). Recent gene flow was ruled out with high confidence in both species (Table S3), further supporting the river hypothesis over the refuge hypothesis. In addition, population expansion analyses found none of the signatures of population bottleneck with subsequent expansion that would be expected under the refuge hypothesis, and strongly rejected this hypothesis in favor of equilibrium isolation‐by‐distance in all of our populations.

In light of the Miocene/Pliocene divergence times and lack of gene flow between these five clades, it is likely that they represent distinct evolutionary lineages. Based on these molecular results, significant differences in scale counts, and even venom toxicity, the Central African populations of *T*. *blandingii* (east of the Congo/Ubangi Rivers) and *T*. *pulverulenta* (east of the Niger Delta) represent cryptic new species that are in the process of being formally described (Greenbaum et al. in press). The two Central African clades of *T. pulverulenta*, separated by the Congo River will be conservatively described as one species based on the results of our STRUCTURE v. 2.3.4 analysis (Falush et al., 2003; Pritchard et al., 2000).

### 4.5 Paleo‐distributions and habitat stability

The nature of the intervening habitat surrounding rainforest refugia during the Pleistocene has been widely debated (reviewed in Couvreur et al., 2021). Some authors have argued that much of the Central African rainforest was replaced by savannas (DeMenocal, 2004; Maley, 1996; Maley & Brenac, 1998), whereas others have emphasized the possibility of more subtle shifts in forest composition (i.e., from wet to dry forest; Colinvaux et al., 1996, 2001; White, 1981). A shift from rainforest to warm temperate woodland and temperate broadleaf evergreen forest, as opposed to savannas, during the last glacial maximum was strongly supported by recent, comprehensive global vegetation models (Allen et al., 2020).

*Toxicodryas* species are generally characterized as arboreal across rainforest and woodland habitats and the two species exhibit widely overlapping distributions in West and Central Africa (Chippaux & Jackson, 2019). Our paleo‐distribution modeling suggested that no substantial contraction of suitable climate occurred for either species during the LGM (Figure 6a), and our habitat stability mapping suggested that core ranges of both species have remained stable for the past 22,000 years (Figure 6c). The greatest potential for habitat expansion in this species appears to be to the south into northern Angola and southern DRC (Figure 6).

Similar paleo‐distribution studies on frogs have suggested substantial habitat contraction in Central Africa during the Pleistocene (Leaché et al., 2019; Portik et al., 2017). In contrast, our inferred widespread habitat stability in *Toxicodryas* may be a result of the relatively reduced dependence of arboreal snakes on moist habitats, as reflected by their distribution in both woodland and rainforest. The stability of *Toxicodryas* habitat through the Pleistocene supports the hypothesis that rainforest composition shifted to dryer woodlands surrounding rainforest refugia, instead of a more dramatic shift to strict savanna habitat. Southward shifts in species suitability may correspond with predicted forest distribution shifts of White (1981) and Allen et al. (2020), suggesting a replacement of lowland rainforest with cooler, more temperate forest.

## 5 CONCLUSION

The complexity of geographic barriers in West and Central Africa, and the association of refugia with areas of high surface relief or riparian zones (Hofer et al., 1999, 2000; Figure 1), makes it extremely difficult to untangle the relative importance of different diversification mechanisms with distribution data alone (Leaché et al., 2019; Portik et al., 2017). This difficulty is particularly salient in our study system, where distribution data could suggest the association of populations with hypothesized refugia around the Congo River, Gabon, and in West Africa (refugia 9, 5–8, and 1–3, respectively, Figures 1 and 3). Yet, our dated phylogenies and paleo‐distribution models reject the Pleistocene population age and habitat contraction predictions of the refugial hypotheses in favor of the river‐barrier hypothesis.

It is important to note, however, that as a result of the difficulty in finding and obtaining tissues from arboreal, venomous, Central African snakes, the small sample sizes used in this analysis may have influenced the inference of our population demographic parameters (Hale et al., 2012; Nelson et al., 2012; Subramanian, 2016; Zwickl & Hillis, 2002). These impacts would have most likely led to an underestimation of the effects of more recent events in the population's history by missing alleles present at low frequencies (Hale et al., 2012; Nelson et al., 2012; Subramanian, 2016). While similar sample sizes have been used in phylogenomic studies of other difficult‐to‐find or difficult‐to‐sample organisms (e.g., Frugone et al., 2019; Muniz et al., 2018; Nash et al., 2018), and simulation studies on empirical data have suggested that small sample sizes (as small as three to four samples per population) can give accurate results if the number of SNPs in the data set is large (Jeffries et al., 2016; Landguth et al., 2012; Nazareno et al., 2017; Qu et al., 2020), more dense sampling of the genus *Toxicodryas* from throughout their range would be ideal for fully understanding the demographic history of these species.

These results highlight the importance of using an integrative, multidisciplinary approach to statistically distinguish among competing hypotheses to explain high levels of geographically concentrated species biodiversity. Moving beyond pure pattern‐based inference, a deeper and more nuanced understanding of the production, partitioning, and maintenance of diversity in complex landscapes may lead to inference of environmental and evolutionary processes that accumulate terrestrial biodiversity in tropical areas, which coincide in many cases with Global Biodiversity Conservation Hotspots (Hrdina & Romportl, 2017; Mittermeier et al., 2000, 2011; Myers, 1988) and other imperiled ecosystems on Earth.

## CONFLICTS OF INTEREST

None declared.

## AUTHORS' CONTRIBUTIONS

K.E.A., A.T.P., and R.M.B. designed the study. E.G., C.K., M.‐O. R, and J.P. provided tissues. K.E.A., P.M.H. and V.V.S. performed the laboratory work. K.E.A., P.M.H and W.P.T.N analyzed the data. K.E.A wrote the manuscript. E.G., P.M.H., J.P., M.‐O. R and R.M.B. edited the manuscript.

## Supporting information

Supplementary Material

## Data Availability

DNA sequences of c‐mos and cyt *b* are accessioned on GenBank (accession numbers: MW655833–MW655873). Data from ddRAD processing, bioclimatic data, and Maxent input files will be re‐archived in the Open Science Framework Digital Repository at https://doi.org/10.17605/OSF.IO/8EC39
