# Supplementary material for: Rivers, not refugia, drove diversification in arboreal, sub‐Saharan African snakes
Source: Ecol Evol. 2021 May 1;11(11):6133–52. doi: 10.1002/ece3.7429 (PMC8207163; doi:10.1002/ece3.7429)
Supplement: Supplementary file 1 — Supplementary Material [file ECE3-11-6133-s001.docx]

**Supporting Information**

Table S1. Museum accession numbers, GenBank accession IDs, and sampling localities for all specimens used in this study.

| Museum Number | Species | Latitude | Longitude | c-mos | cyt *b* |
| --- | --- | --- | --- | --- | --- |
| LSUMZ 20224 | *Toxicodryas blandingii* | 7.861808 | -1.593136 | MW655852 | MW655869 |
| PB11 640 | *Toxicodryas blandingii* | 10.3801 | -9.300244 | MW655850 | MW655870 |
| CAS 253611 | *Toxicodryas blandingii* | 4.4732 | 11.9648889 | MW655847 | MW655868 |
| UTEP 22193 | *Toxicodryas blandingii* | -2.58617 | 16.47339 | MW655849 | MW655872 |
| UTEP 22195 | *Toxicodryas blandingii* | -1.878 | 28.4524 | MW655848 | MW655871 |
| UTEP 22194 | *Toxicodryas blandingii* | -4.71209 | 19.4802 | MW655851 | MW655873 |
| CAS 258155 | *Toxicodryas pulverulenta* | -2.2419 | 13.58746 | MW655834 | MW655856 |
| MCZ 187704 | *Toxicodryas pulverulenta* | 0.51624 | 12.79457 | MW655836 | MW655858 |
| USNM 584253 | *Toxicodryas pulverulenta* | -2.612499 | 13.615022 | MW655845 | N/A |
| CAS 254591 | *Toxicodryas pulverulenta* | -1.10775 | 10.02691 | MW655853 | MW655854 |
| CAS 253375 | *Toxicodryas pulverulenta* | 2.6106 | 14.0234 | MW655833 | MW655855 |
| CAS 258156 | *Toxicodryas pulverulenta* | -2.2419 | 13.58746 | MW655846 | MW655857 |
| ZMB 86412 | *Toxicodryas pulverulenta* | 7.755777 | -8.814877 | MW655843 | MW655867 |
| ZMB 86410 | *Toxicodryas pulverulenta* | 7.575188 | -9.242767 | MW655842 | MW655864 |
| ZMB 86414 | *Toxicodryas pulverulenta* | 7.585033 | -9.224848 | MW655844 | MW655866 |
| ZMB 86384 | *Toxicodryas pulverulenta* | 7.755777 | -8.814877 | MW655841 | MW655865 |
| UTEP 22205 | *Toxicodryas pulverulenta* | -1.14679 | 20.80005 | MW655835 | MW655863 |
| UTEP 22200 | *Toxicodryas pulverulenta* | -4.16473 | 28.16529 | MW655838 | MW655860 |
| UTEP 22197 | *Toxicodryas pulverulenta* | -3.24329 | 24.24856 | MW655837 | MW655859 |
| UTEP 22201 | *Toxicodryas pulverulenta* | -4.08432 | 28.15373 | MW655839 | MW655862 |
| UTEP 22203 | *Toxicodryas pulverulenta* | -0.44791 | 18.1346 | MW655840 | MW655861 |
|  | *Dasypeltis atra* |  |  | AF471136 | AF471065 |
|  | *Dasypeltis scabra* |  |  | AY611945 | AY612036 |
|  | *Contia tenuis* |  |  | AF471134 | AF471095 |
|  | *Heterodon simus* |  |  | AF471142 | AF217840 |
|  | *Hemachatus haemachatus* |  |  | N/A | AF217821 |
|  | *Atheris nietschei* |  |  | AF471125 | AF471070 |
|  | *Naja Kaouthia* |  |  | AY058938 | AF217835 |
|  | *Crotaphopeltis hoetamboeia* |  |  | AY611973 | AY612064 |
|  | *Crotaphopeltis tornieri* |  |  | AF471112 | AF471093 |
|  | *Dasypeltis medici* |  |  | AY611990 | AY612081 |
|  | *Dasypeltis fasciata* |  |  | KX660328 | KX660463 |
|  | *Dipsadoboa unicolor* |  |  | AF471139 | AF471062 |

Table S2. DelimitR confusion matrices. Red indicates the number out of 100,000 simulated data sets that were correctly classified by the random forest classifiers, orange and yellow indicate the number of models that were incorrectly classified with yellow indicating few to no misclassified models and orange indicating a higher number of misclassified models. Model 1: no divergence, model 2: divergence without gene flow, model 3: divergence with secondary contact, and model 4: divergence with gene flow.

| ***T. blandingii*** | Model 1 | Model 2 | Model 3 | Model 4 |
| --- | --- | --- | --- | --- |
| Model 1 | 91990 | 0 | 8010 | 0 |
| Model 2 | 0 | 87719 | 13 | 12268 |
| Model 3 | 36659 | 39 | 63201 | 101 |
| Model 4 | 0 | 11847 | 160 | 87993 |

| ***T. pulverulenta*** | Model 1 | Model 2 | Model 3 | Model 4 |
| --- | --- | --- | --- | --- |
| Model 1 | 89710 | 0 | 10290 | 0 |
| Model 2 | 0 | 81778 | 39 | 18183 |
| Model 3 | 45225 | 48 | 54520 | 207 |
| Model 4 | 2 | 17002 | 329 | 82667 |

_­_

Table S3. The number of 500 random forest classifiers that voted for each competing demographic model in delimitR. Model 1: no divergence, model 2: divergence without gene flow, model 3: divergence with secondary contact, and model 4: divergence with gene flow.

|  | Model 1 | Model 2 | Model 3 | Model 4 |
| --- | --- | --- | --- | --- |
| *T. blandingii* | 1 | 278 | 6 | 215 |
| *T. pulverulenta* | 0 | 150 | 81 | 269 |


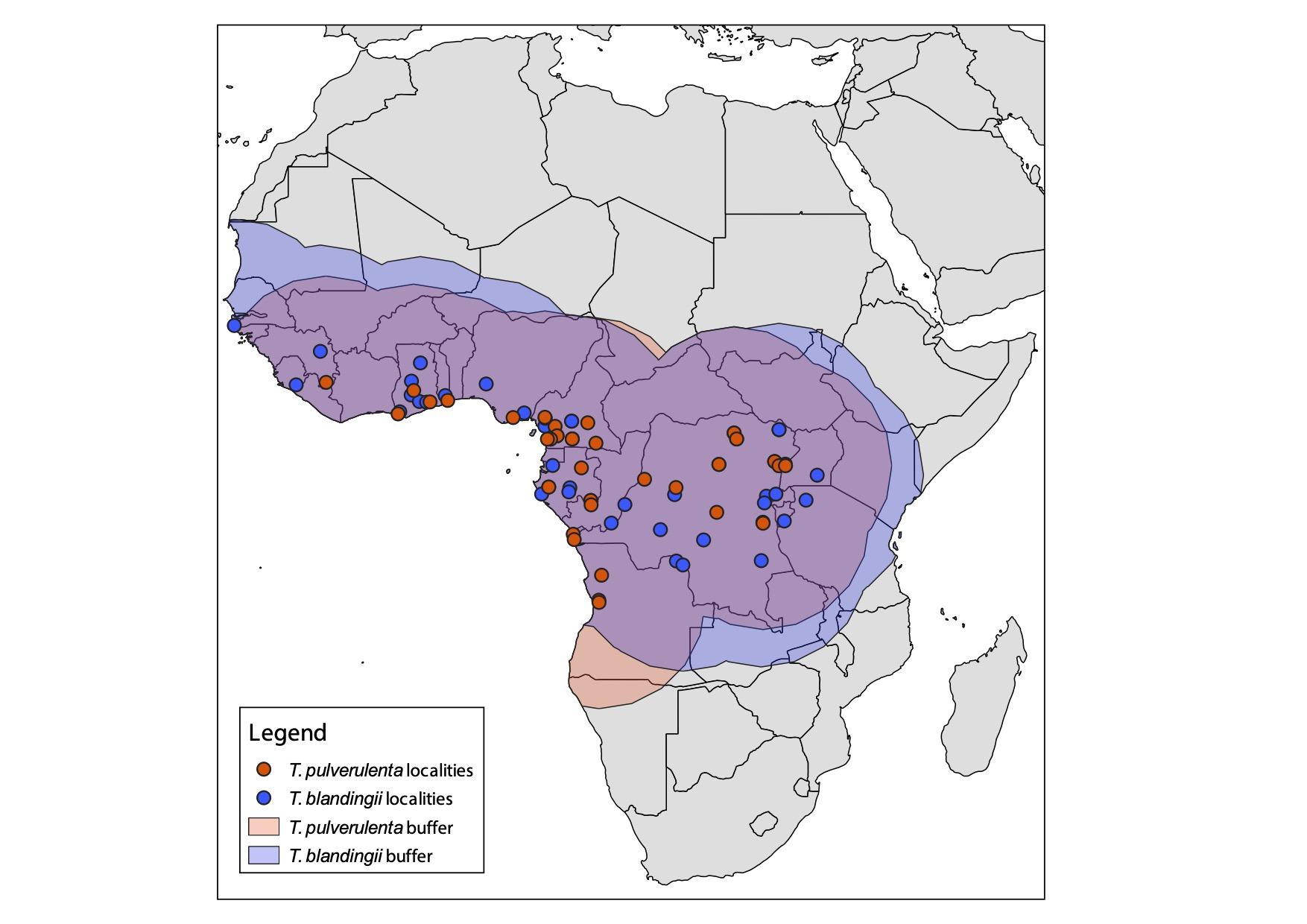


Figure S1. Points and 1000-kilometer buffers used for niche modeling parsed by species

B

A

Figure S2. Phylogenetic trees estimated from full ddRADseq data sets in A) SVDquartets, and B) IQtree. Numerical nodal support values represent percentages of 100 nonparametric bootstrap replicates in (A) and percentages of 10,000 ultrafast bootstrap replicates in (B). Branch lengths in (B) are proportional to expected substitutions per site.


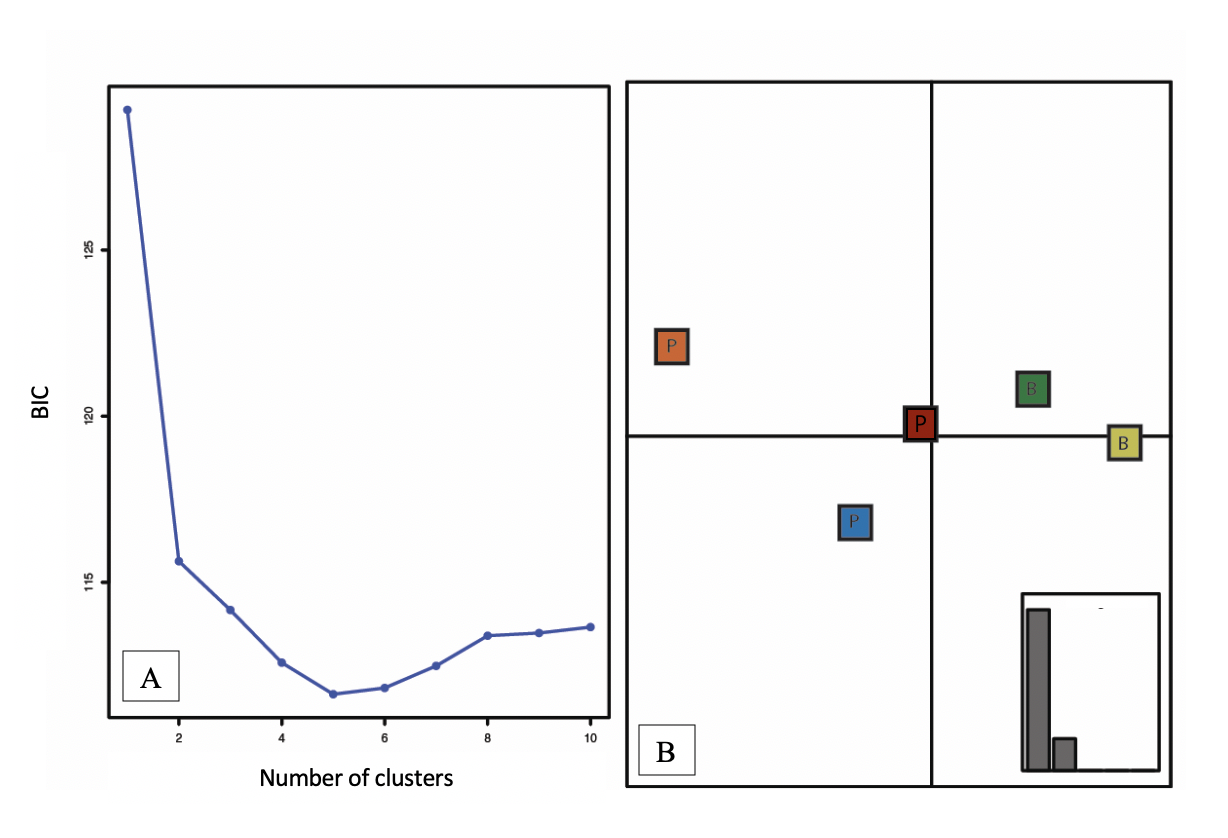


Figure S3. DAPC population structure analysis. A) inference of population number using BIC suggests 5 genetic clusters and B) scatterplot for those 5 clusters and the barplot of the DA eigenvalues. Colors correspond to clades in Figure 2.
